# Neuraminidase-3 activity in toddlers negatively impacts the linear growth status in Bangladeshi children

**DOI:** 10.1038/s41598-025-12763-4

**Published:** 2025-07-24

**Authors:** Subhasish Das, Visnu Pritom Chowdhury, Md Amran Gazi, Md Golam Rasul, Richard J. Roberts, Tahmeed Ahmed

**Affiliations:** 1 Nutrition Research Division, International Centre for Diarrhoeal Disease Research, Bangladesh (icddr,b), Dhaka, Bangladesh; 2 Liggins Institute, University of Auckland, Auckland, New Zealand; 3 Department of Biological Sciences, University of Delaware, Newark, DE USA; 4 Department of Nutritional Sciences, University of Wisconsin-Madison, Madison, WI USA; 5 Department of Global Health, University of Washington, Seattle, Washington USA; 6 Department of Public Health Nutrition, James P. Grant School of Public Health, BRAC University, Dhaka, Bangladesh

**Keywords:** Biomarkers, Predictive markers

## Abstract

Linear growth faltering and stunting in children is associated with inflammation of small gut. Intestinal alkaline phosphatase prevents gut inflammation from subclinical bacterial infection that becomes impaired in the presence of neuraminidase-3 (Neu3) activity. We investigated if total Neu3 in children at 15 months can predict their linear growth at later ages (at 18, 21, and 24 months). We collected data from 189 children enrolled in the Malnutrition and Enteric Disease Study (MAL-ED) birth cohort study in Bangladesh. We determined total Neu3 activity in stool samples at 15 months of age and measured its association with length-for-age z-scores (LAZ) at 18, 21, and 24 months of age, in addition to their socio-demographic conditions using bi-variate and multivariable linear regression analyses. We found that total Neu3 at 15 months was negatively associated with the LAZ-score at 18 (regression coefficient -0.004, 95% CI -0.006 to -0.001, p < 0.01), 21 (-0.003, 95% CI -0.006, -0.001, p < 0.01), and 24 (-0.004, 95% CI -0.006, -0.001, p < 0.01) months of age after adjusting the covariates. In conclusion, total neuraminidase-3 in stool is a significant predictor of linear growth in young children and would be key in early detection of linear growth faltering and stunting.

## Introduction

Stunting is a condition where a child’s height falls below two standard deviations of the World Health Organisation (WHO) Child Growth Standard^1^. According to the 2020 WHO report, 149.2 million children under the age of five years were stunted^2^. Despite improvement in the last few decades, the burden of stunting in developing countries is still alarming^3^. Stunting was thought to be caused by undernutrition and poor hygiene, however, in Bangladesh, a recent report showed that 21% of children from the privileged part of the population were stunted as well^4^. Stunting is also associated with an increased risk of developing learning deficits (due to poor cognitive development) as well as non-communicable diseases like diabetes and hypertension^5,6^. Complications caused by stunting are irreversible if not addressed quickly^7^. Thus, stunting is an issue that impacts young children in all socioeconomic conditions, that requires early intervention to prevent long-term sequelae.

A recent report showed that environmental enteric dysfunction (EED) – a subclinical chronic inflammatory disorder of the small intestine – underlies 40% of all stunting cases^8^, even when diarrhea is controlled^9,10^. EED is still highly prevalent in low- and middle-income countries, especially in children who dwell in slums^8^. During EED the microvilli of the gut epithelium become flattened and the crypts become shallow^11,12^. Also, the intestinal wall loses its capacity to absorb nutrients while simultaneously compromising the barrier function^13^ leading to bidirectional leakage of fluid, pathogens, and toxins across the intestinal wall^11,12,14^. This functional impairment of the gut epithelium is the major driver of the gut inflammation seen in EED cases and may be a mechanism by which EED contributes to stunting. One of the enzymes involved in mitigating this inflammatory response in the gut is intestinal alkaline phosphatase (IAP).

**Fig. 1:**
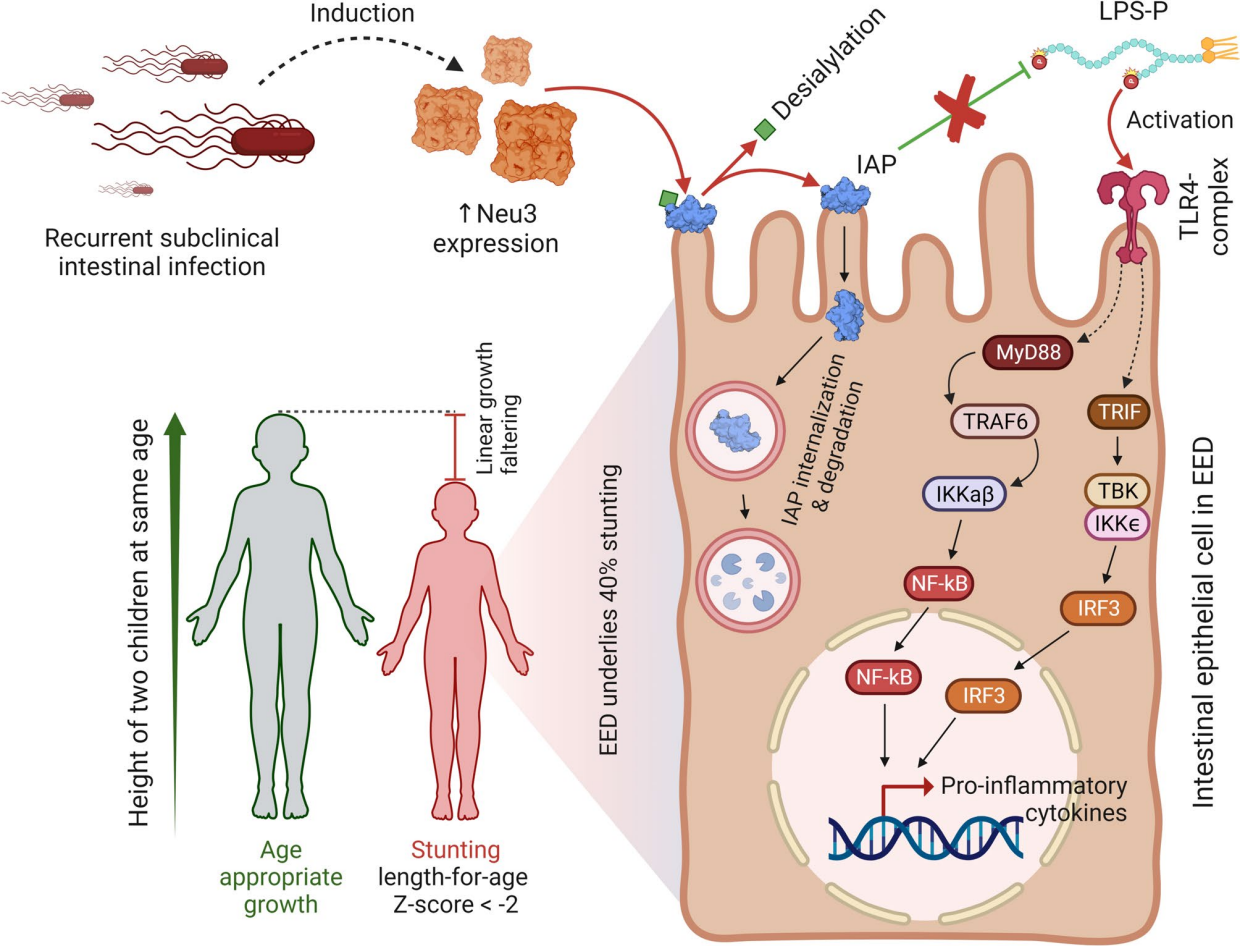
Graphical summary indicating the relationship between stunting and EED. (Stunting is a form of linear growth faltering in children, which is often associated with EED (environmental enteric dysfunction). In EED, the microvilli of the gut epithelium become flattened, and crypts become shallow. Recurrent subclinical enteric infection ensues higher expression of a sialidase, neuraminidase 3 (Neu3). It cleaves off the sialic acid residue from IAP, increasing its internalization and degradation. IAP is capable of inactivating LPS-P and attenuating gut inflammation. However, in the absence of IAP, TLR4-complex becomes activated, which results in the release of pro-inflammatory cytokines, through activation of MyD88-dependent and MyD88-independent signaling pathways 25–28. EED – Environmental Enteric Dysfunction; TLR4—Toll-like receptor 4; Neu3 – Neuraminidase 3; IAP – Intestinal Alkaline Phosphatase; LSP-P – Phosphorylated Lipopolysaccharide; TRIF—TIR-domain-containing adapter-inducing interferon-β; MyD88—Myeloid differentiation primary response 88; IKK—inhibitor of nuclear factor-κB kinase; TBK—TANK-binding kinase; NF-κB—Nuclear factor kappa B; IRF3—IKK, and interferon regulatory factor 3).

IAP is a dephosphorylating enzyme found in the apical brush border of the small intestine. It neutralizes intestinal pathogens and ensures optimal intestinal functionality. Although IAP produced exclusively by the duodenal enterocytes, the activity is highest in the terminal part of the ileum^15–17^. IAP dephosphorylates the lipopolysaccharide-phosphate (LPS-P) endotoxin thereby preventing the activation of the TLR-4-complex mediated gut inflammation^18–21^. However, IAP loss of function (caused by decreased catalytic activity with/without decreased enzyme expression) can result in reduced detoxification of LPS-dependent TLR-4 agonist activity^22^. Activation of TLR4, for example in recurrent subclinical *Salmonella enterica* Typhimurium infection, can lead to Toll-like receptor 4 (TLR4)-dependent expression of neuraminidase-3 (Neu3), triggering the onset of gut inflammation^23^. Although *Salmonella* – a major human foodborne pathogen—does not code for neuraminidase, it is widely encoded by diverse organisms, including the human gut microbiota^23,24^. Neuraminidases, also known as sialidases, is also expressed by human cells and cleaves sialic acid from glycan moieties^25^. Neu3 was reported to trigger intestinal inflammation and colitis in human^23^. An increased neuraminidase activity can hydrolyze the IAP, reducing its half-life and leading to an acquired deficiency^26^. Impaired expression of IAP has also been found to be associated with inflammatory bowel disease and celiac disease^17^. Given the impact of Neu3 on IAP it is worth investigating whether it can be used as a marker to indicate intestinal health and functionality. Figure 1 presents the graphical summary indicating the relationship EED, Neu-3 and stunting.

In this context, we hypothesize that the Neu3 activity in young children will negatively impact their nutritional status, leading to stunting. To test our hypothesis, we measured the total Neu3 in children at 15 months and their linear growth status at the ages of 18, 21, and 24 months, to determine if Neu3 has the potential to be used as a predictor of linear growth.

## Methods

### Study site, study design, participants, and ethical consideration

In this study, we used the data and the biological samples collected from the children of the Malnutrition and Enteric Disease Study (MAL-ED) in Bangladesh. In the MALED study, children enrolled at birth at the Bauniabadh area of Mirpur, Dhaka and the adjacent community were followed up to 24 months of age. The study protocol (ethical approval number: PR-18068) was approved by the Institutional Review Board of International Centre for Diarrhoeal Disease Research, Bangladesh (icddr,b). Informed written consent was taken from one of the parents of each study participant. All the methods were performed following the relevant guidelines and regulations. A detailed description of the MAL-ED study methodology has been published elsewhere^27^.

### Data collection, anthropometry, specimen collection and storage

Baseline sociodemographic and household information of the children was collected at enrollment after birth. Following standard anthropometric methodology, trained field workers measured the recumbent length of the children to the nearest 1 mm using Seca infantometer (model no: 417, Hamburg, Germany) every month until 24 months of age^28^. The length-for-age z-score (LAZ-score) was calculated according to the WHO 2006 child growth standards using the lambda-mu-sigma method^29^. The LAZ-score was used to assess the linear growth status. Length was measured using commercial measuring boards (Seca Infantometer; model no. 417). Stored stool samples from 188 children in the MAL-ED fifteen-month cohort were used to assess the levels of Neu3. Stool samples were collected without a fixative, and were aliquoted and stored at –70 °C. The 15 month time point was chosen as it had the maximum number of stool samples available for analysis.

### Determination of total neuraminidase activity

The total neuraminidase activity was evaluated with the product technical sheet obtained from the neuraminidase activity assay kit of Sigma-Aldrich. Briefly, 480 uL of water was added to 20 uL of the 10 mM standard (standard enzyme, provided with the kit) to make a 400-uM standard working solution. Appropriate volumes of water were added to make 0 (blank), 120, 240, and 400 uM standards. Then 20 µl of standards were added to separate wells of a 96-well clear flat-bottom plate in four replicates. 80 µl of the reaction mixture with substrate was added to 2 wells and 80 µl reaction mixture alone (control) was added to the remaining 2 wells. The plate was sealed and incubated at 37 °C in the dark. Absorbance was measured at 570 nm at 20 min and 50 min. Absorbance measured at 50 min was plotted for each standard against the standard concentrations. The linearity of standard curve was evaluated, slope of the linear regression was determined, and the activity of each sample was calculated according to the manufacturer’s protocol.

### Explanatory and outcome variables

Mean values of morbidity (diarrhea and fever) episodes and total calorie intake during the preceding three months of assessment of outcome variables were used as indicators of morbidity and energy intake. Trained field workers visited the participant’s household twice a week and used a surveillance assessment form (SAF) to document illness status of the child. During every such visits, field workers asked the caregiver whether the children experienced any morbidity symptoms listed in SAF on each day since the last visit. Diarrhoea is defined as having three or more loose stools in a 24-h period or at least one loose stool with blood reported by the mother. A diarrhoeal episode is defined as being separated from another episode by at least two or more diarrhoea-free days. Fever is defined as an axillary temperature > 37.5 °C^30^. The 24-h food frequency data were collected monthly from ninth month onwards using a 24-h multiple-pass dietary recall approach for assessing the energy intake of the children. The 24-h dietary recall interviews were conducted on non-consecutive days and out of every four recalls, one interview was conducted on a weekend. A locally adapted food composition table was used to convert the dietary intake data to energy^31^. WAMI index (Water, sanitation, hygiene, Asset, Maternal education, and Income index, ranging from 0 to 1) is a socioeconomic status index that includes access to improved water and sanitation, eight selected assets, maternal education, and household income was used as a representative of socio-economic status of the households^32^. A higher WAMI index means a better socioeconomic status. The composite environmental enteric dysfunction score or EED score ranging from 0 to 10 was calculated at 15 months from alpha-1-anti-trypsin, myeloperoxidase and neopterin- the three fecal markers of environmental enteric dysfunction following the method published previously^33^.

### Statistical analyses

Secondary analyses of MAL-ED birth cohort study data was conducted. Socio-economic characteristics and demographic factors were described using frequencies with proportions when variables were categorical. Means and standard deviations (SD) were reported when quantitative variables were distributed normally. Medians and interquartile ranges (IQRs) were used when the quantitative variables were not normally distributed. Correlations between the Neu3 abundance and LAZ scores at 18, 21, and 24 months were assessed using Spearman’s correlation test. Association of Neu3 and other predictors to LAZ scores at 18, 21, and 24 months were measured using bivariable and multivariable linear regression models. At first, bivariable analysis was done to explore the unadjusted effect of Neu3, morbidity, socio-economic status, total calorie intake, and birth characteristics on the outcomes. Variables were assessed individually and were included in the multivariable regression model if the p-value was found < 0.20 in bivariable analysis following the recommendation of Greenland et al.^34^. A p-value < 0.05 was considered statistically significant for multivariable regression analyses. Analyses were performed using Stata version 18 and R version 3.5.3^35^.

## Results

Table 1 shows the characteristics of the 189 participants in this study. Half of them were males. The length for age (LAZ) scores of the cohort decreased slightly between 18, 21 and 24 months.

**Table 1:** Characteristics of the participants.

Basic Demographic Information	
Male, n (%)	84 (48.55)
WAMI index*, Mean (SD)	0.537 (0.128)
Maternal Height, Mean (SD), cm	149.293 (4.75)
Birth weight, Mean (SD), kg	2.794 (0.416)
Exclusive breastfeeding days, Median (IQR)	104 (96)
Neu3 protein abundance at 15 months, Median (IQR), ng/ml	30.78 (46.78)
EED Score**, Median (IQR), (at 15 months)	5 (3)
LAZ-score, Mean (SD)	At month 18: −1.93 (0.91)
	At month 21: −1.99 (0.92)
	At month 24: −2.00 (0.91)
Diarrhea episode, Median (IQR)	15–17 months: 0.33 (0.33)
	18–20 months: 0.33 (0.66)
	21–23 months: 0.25 (0.33)
Fever episode, Median (IQR)	15–17 months: 0.66 (0.66)
	18–20 months: 0.66 (0.66)
	21–23 months: 0.50 (0.50)
Total calorie intake, Median (IQR)	15–17 months: 309.38 (178.69)
	18–20 months: 391.81 (197.90)
	21–23 months: 483.20 (243.23)

*WAMI index: WAMI index (Water, sanitation, hygiene, Asset, Maternal education, and Income index, ranging from 0 to 1) is a socioeconomic status index that includes access to improved water and sanitation, eight selected assets, maternal education, and household income was used as a representative of socio-economic status of the households. **EED score: The composite environmental enteric dysfunction score or EED score ranging from 0 to 10 was calculated from alpha-1-anti-trypsin, myeloperoxidase and neopterin- the three fecal markers of environmental enteric dysfunction.

**Fig. 2:**
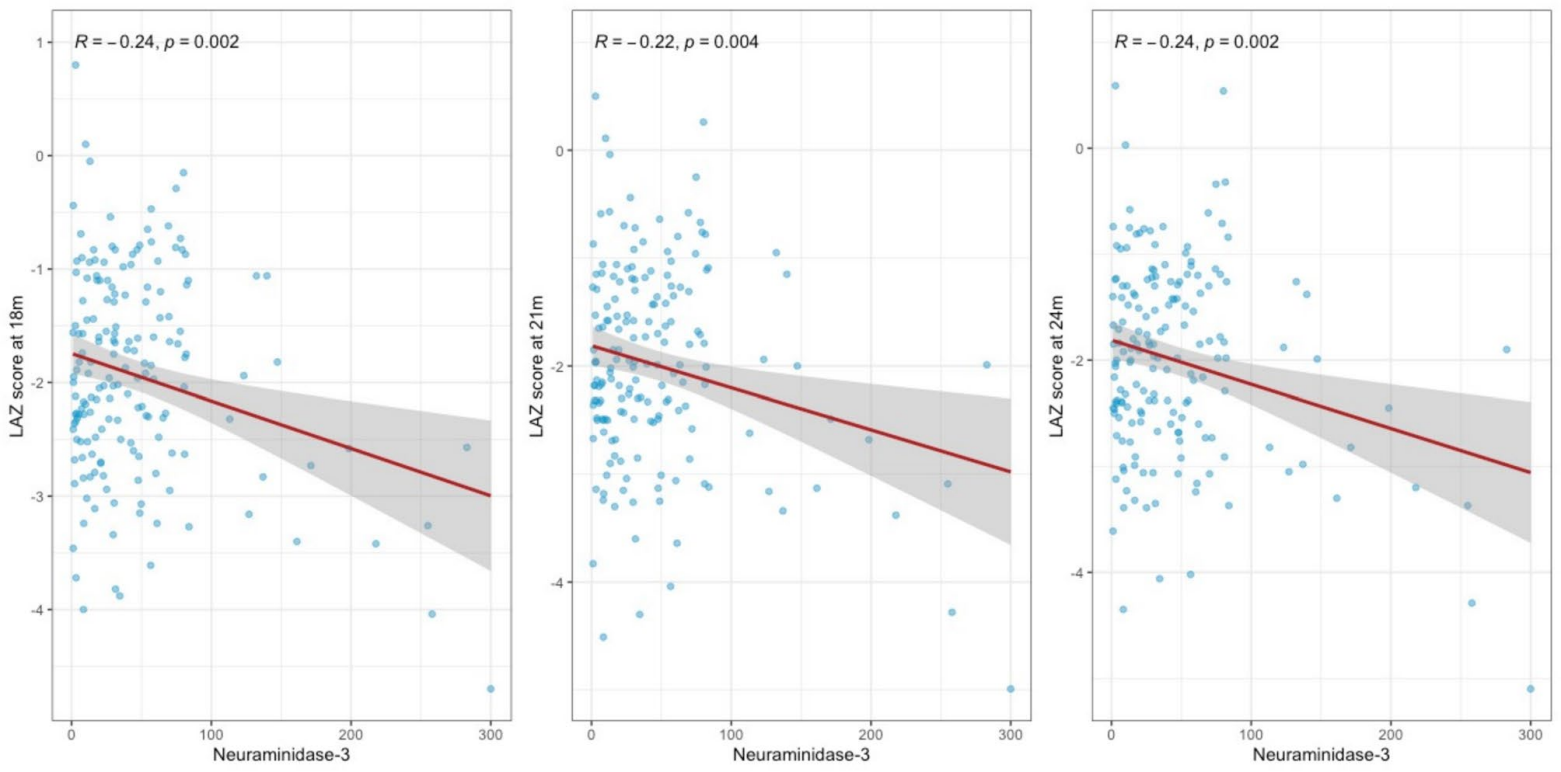
The correlation matrix of Neu3 levels at 15 months and LAZ-scores at 18, 21, and 24 months.

Figure 2 presents the correlation matrix of Neu3 levels at 15 months and LAZ-scores at 18, 21, and 24 months. Data shows that the Neu3 level at 15 months had a weak, yet statistically significant negative correlation with LAZ-scores at subsequent months.

Table 2 shows the associations between Neu3 and other predictors with LAZ scores at different time points. Our findings indicate that LAZ scores at 18, 21, and 24 months were significantly associated with the total Neu3 levels measured at 15 months of age, WAMI index, maternal height, and birth weight of the children. However, the total calorie intake status showed a statistically significant association with LAZ-score only at 18 months of age.

**Table 2:** Association of Neu3 and other predictors to LAZ-score at 18, 21, and 24 months: results from bivariable linear regression analyses.

	LAZ-score at month 18		LAZ-score at month 21		LAZ-score at month 24	
	Coefficient (95% CI)	P-value	Coefficient (95% CI)	P-value	Coefficient (95% CI)	P-value
Neu3	−0.004 (−0.007, −0.002)	< 0.01	−0.004 (−0.006, −0.001)	< 0.01	−0.004 (−0.007, −0.002)	< 0.01
Diarrhea episode	−0.190 (−0.595, 0.214)	0.36	0.112 (−0.282, 0.506)	0.58	−0.152 (−0.542, 0.238)	0.45
Fever episode	−0.150 (−0.419, 0.118)	0.27	0.209 (−0.098, 0.516)	0.18	−0.166 (−0.514, 0.183)	0.35
Total calorie intake	0.001 (0.000, 0.002)	0.05	0.001 (0.000, 0.001)	0.10	0.000 (0.000, 0.001)	0.39
Gender	−0.114 (−0.386, 0.158)	0.41	−0.027 (−0.303, 0.250)	0.85	−0.111 (−0.383,0.161)	0.42
WAMI index	2.149 (1.126, 3.172)	< 0.001	2.440 (1.413, 3.466)	< 0.001	2.161 (1.137, 3.185)	< 0.001
Mother’s height	0.061 (0.034, 0.088)	< 0.001	0.066 (0.038, 0.093)	< 0.001	0.069 (0.043, 0.096)	< 0.001
Birthweight	0.829 (0.526, 1.133)	< 0.001	0.871 (0.564, 1.178)	< 0.001	0.776 (0.468, 1.083)	< 0.001
Exclusive breastfeeding days	−0.001 (−0.003, 0.002)	0.52	−0.001 (−0.003, 0.001)	0.38	−0.001 (−0.003, 0.002)	0.54
EED score	−0.005 (−0.071, 0.061)	0.89	0.007 (−0.061, 0.074)	0.84	−0.001 (−0.068, 0.065)	0.97

In the multivariable linear regression model, we investigated the adjusted associations of LAZ with biomarkers and socio-demographic characteristics. Table 3 presents the independent variables (p-value ≤ 0.05) included in multivariable analysis. We found that after adjusting for all significant independent variables, total Neu3, maternal height, and birth weight of the children remained significantly associated with the LAZ-score at all three time points. However, the WAMI index was significantly associated at 21 and 24 months.

**Table 3:** Association of Neu3 and other predictors to LAZ-score at 18, 21, and 24 months: results from multivariable linear regression analyses.

	LAZ-score at month 18		LAZ-score at month 21		LAZ-score at month 24	
	Coefficient (95% CI)	P-value	Coefficient (95% CI)	P-value	Coefficient (95% CI)	P-value
Neu3	−0.004 (−0.006, −0.001)	< 0.01	−0.003 (−0.006, −0.001)	< 0.01	−0.004 (−0.006, −0.001)	< 0.01
Total calorie intake	0.000 (−0.001, 0.001)	0.47	-	-	-	-
WAMI index	0.998 (−0.004, 2.000)	0.05	1.360 (0.387, 2.332)	< 0.01	1.067 (0.100, 2.035)	0.03
Mother’s height	0.041 (0.016, 0.067)	< 0.01	0.044 (0.018, 0.069)	< 0.01	0.051 (0.025, 0.076)	< 0.001
Birthweight	0.675 (0.384, 0.966)	< 0.001	0.698 (0.407, 0.989)	< 0.001	0.610 (0.321, 0.900)	< 0.001

## Discussion

Previous studies indicate that a child’s weight and length at birth, maternal nutritional status, and parents’ height are some of the strongest biological predictors of linear growth ^36,37^. However, our data shows in addition to the maternal height and birthweight, total neuraminidase-3 at 15 months of age is a predictor of liner growth at later stages, i.e., at 18, 21, and 24 months.

The finding of Neu3 as a predictor of linear growth has biological relevance. Although Neu3 could be endogenous (encoded in chromosome 11 in humans)^38^, neuraminidases are produced by various pathogens^39,40^. Thus, a higher level of Neu3 in children suggests possible subclinical infection with such pathogens. Previous studies showed the induction of Neu3 caused severe inflammation in both small and large intestines in animal models^23^. Other studies supported this finding by showing that Neu3 can activate neutrophils, which are inducers of inflammation^41^. It is also known that Neu3 impairs the activity of intestinal alkaline phosphatase, which is critical for neutralizing microbial lipopolysaccharide. In the absence of IAP, LPS activates toll-like receptor 4 complex and mediates gut inflammation^42^, which damages the gut epithelium, causes the infiltration of the gut microbes, activates the local immune cells^43^, leading to further damage, malabsorption, and malnutrition^44,45^. All these point to the direction of Neu3, indicating this enzyme is one of the key factors mechanistically involved in malnutrition.

In addition to that, socio-economic factors, including maternal education, sanitation and hygiene practice, economic status of the household, also impact the growth and development of children^46^. Our data also indicate that socio-economic factors, such as better-quality water, sanitation, maternal education, and higher household income (combined as the WAMI index) can impact children’s linear growth. However, our data suggests that the WAMI factor does not impact the length of a child as early as 18 months of age. It means total Neu3 might be a key predictor at the of linear growth earlier stages of development.

Here, we measured the total neuraminidase-3 in stool in the enrolled children. We could not determine whether the Neu3 is endogenous or exogenous^39,47,48^. Determining the source of Neu3 would help formulate the appropriate strategies needed to ensure maximum growth and development in children. The samples we collected from patients are from the Bauniabadh area of Mirpur, Dhaka^49^, a well-known resource-limited urban area in Bangladesh. Even though our sample size is reasonable, having more samples from other parts of this country is required to determine if our findings are generalizable. Previous studies indicate that approximately 40% of stunting is explained by EED, which is one form of chronic inflammatory bowel disorder^8^. Chronic inflammation in EED suggests lower inhibition of inflammation that could be associated with IAP induced modulation Neu-3 pathways. However, more longitudinal analyses are required to elucidate the role of Neu3 as a predictor or biomarker of EED, liner growth, and stunting. Our current findings demonstrate that use of inhibitors for Neu-3 could be implicated in stunting. This means very likely there are other biological modifiers that impact stunting, still waiting to be discovered.

In conclusion, we found a novel predictor of childhood linear growth, total neuraminidase 3, which can be used as a predictor of linear growth and help clinicians plan a realistic and optimal course of nutritional intervention so that a child can catch up with the linear growth faltering and reach full potential.

## Data Availability

The dataset generated and analyzed during the current study is available from the corresponding author on reasonable request.
